# Severe Tricuspid Regurgitation in a Patient With Previous Tricuspid Valve Surgery for Infective Endocarditis Secondary to Intravenous Drug Use: A Case Report

**DOI:** 10.7759/cureus.40497

**Published:** 2023-06-16

**Authors:** Zahid Khan, Amresh Gul, Gideon Mlawa

**Affiliations:** 1 Acute Medicine, Mid and South Essex NHS Foundation Trust, Southend-on-Sea, GBR; 2 Cardiology, Barts Heart Centre, London, GBR; 3 Cardiology and General Medicine, Barking, Havering and Redbridge University Hospitals NHS Trust, London, GBR; 4 Cardiology, Royal Free Hospital, London, GBR; 5 General Practice, Lifeline Hospital, Salalah, OMN; 6 Internal Medicine and Diabetes and Endocrinology, Barking, Havering and Redbridge University Hospitals NHS Trust, London, GBR

**Keywords:** culture-negative infective endocarditis, right-sided infective endocarditis, blood culture-negative endocarditis, tricuspid valve repair, heroin use disorder, intravenous drug use (ivdu), moderate to severe mitral regurgitation, biventricular dysfunction, flail tricuspid valve, tricuspid valve endocarditis

## Abstract

Tricuspid regurgitation (TR) is an important but underappreciated disease in medical practice, and the severity can vary from moderate to severe. Right-sided infective endocarditis (RSIE) is more common in intravenous drug users (IVDUs), and the vast majority of these involve the tricuspid valve (TV). It is worth mentioning that right-sided valves are challenging to scan compared to left-sided valves. The incidence of severe tricuspid regurgitation (TR) immediately post-repair is not tangible, but it is considered to be rare. We present a case of a 47-year-old patient who had previous TV septal leaflet reconstruction using a bovine pericardial patch using 6/0 prolene, and an annuloplasty was performed by placing an annuloplasty ring in 2017 for infective endocarditis. The patient developed moderate to severe tricuspid regurgitation within a few weeks following the surgery. She was readmitted to the hospital four years later with a reduced consciousness level, and a subsequent repeat echocardiogram showed possible tricuspid valve vegetation. In addition, transoesophageal echocardiogram (TOE) demonstrated biventricular dysfunction and severe tricuspid regurgitation, along with moderate to severe mitral regurgitation (MR) that was variable depending on the rate of atrial fibrillation. The patient was not suitable for surgical intervention and was medically managed accordingly.

## Introduction

Tricuspid valve (TV) endocarditis is an uncommon presentation compared to left-sided endocarditis and accounts for about 5%-10% of total infective endocarditis (IE) cases [1]. The vast majority of right-sided IE (RSIE) cases involve TV, and it is most common in patients with intravenous drug use (IVDU) [1]. It is believed that about 90% of RSIE involve TV [1]. In developed countries, it is the most common cause of acute tricuspid regurgitation (TR) [1]. In a study by Oxford University, the prevalence of TVE ranged from 5% to 10% of cases of heart valve endocarditis (HVE) [2,3]. Risk factors include intravenous drug use, cardiac device implant, central venous catheters, HIV, and congenital heart disease (CHD) [4,5].

*Staphylococcus aureus* is the most common organism that causes tricuspid valve infective endocarditis. However, various skin flora, as well as various *Staphylococcus* and *Streptococcus* species, can infect the tricuspid valve. Duke’s criteria are used to establish the diagnosis of tricuspid valve infective endocarditis (TVIE). The leading bacterial cause of infective endocarditis (IE) is *Staphylococcus aureus*, followed by *Streptococcus viridans*, with rare presentations by *Staphylococcus epidermidis*, *Streptococcus bovis*, and *Haemophilus* species, *Aggregatibacter* species, *Cardiobacterium hominis*, *Eikenella corrodens*, and *Kingella* species (HACEK) organisms [6]. There are certain aspects of TVIE that can make its detection difficult, such as the absence of murmur, concurrent pneumonia, and less prominent peripheral signs, including splinter hemorrhage, Osler nodes, and Janeway lesions [7]. RSIE and TVIE are increasing in prevalence, and antibiotics and surgical options remain a cornerstone of successful treatment. We present a case of a 47-year-old patient with TVIE secondary to IVDU who developed severe TR and biventricular impairment.

## Case presentation

A 47-year-old female patient presented to the accident and emergency (A&E) department with disorientation, irritability, and confusion for the last week. On admission, she was in atrial fibrillation with a rapid ventricular response of 191 beats per minute, blood pressure of 104/77 mmHg, respiratory rate (RR) of 18 per minute, 98% SpO2 on room air, and temperature of 36.2°C. She appeared cachectic, drowsy, and confused, had poor dental hygiene, and had a pansystolic murmur in the fourth intercostal space on auscultation. She attended the hospital about a fortnight ago but self-discharged after five days of hospital admission. She represented to the A&E department 10 days later with confusion, irritability, and fatigue for about a week. She was initially treated for possible TVIE in view of her past medical history and recent echocardiogram showing possible TV vegetation prior to her discharge against medical advice on the last admission.

Her past medical history was significant for tricuspid valve reconstruction and annuloplasty in 2017 following infective endocarditis, chronic obstructive pulmonary disease (COPD), hepatitis C virus (HCV), and recreational intravenous drug use (IVDU). Her previous TV surgery in 2017 included tricuspid valve septal leaflet reconstruction with a pericardial bovine patch, and an annuloplasty was performed by placing an annuloplasty ring following diagnosis with group G streptococcus infective endocarditis. A repeat echocardiogram a week following TV surgery showed moderate to severe TR with normal biventricular function in 2017. She smoked five bags of cracked cocaine and three bags of heroin overnight for the last several years. She consumed an unclear quantity of alcohol.

Her initial blood report showed raised potassium, hyponatremia, deranged liver enzymes, and very mildly raised C-reactive protein (CRP) as shown in Table 1.

**Table 1:** Laboratory values of the patient during hospital admission Hb: hemoglobin, WCC: white cell count, ESR: erythrocyte sedimentation rate, CRP: C-reactive protein, K: potassium, Na: sodium, Ca: calcium, Cr: creatinine, eGFR: estimated glomerular filtration rate, ALT: alanine transaminase, ALP: alkaline phosphatase, INR: international normalized ratio

Blood test	Day 1	Day 3	Day 4	Day 7	Day 9	Day 15	Normal range
Hb (g/L)	159	157	138	153	180	153	120-150
WCC (×10^9^/L)	10	11	13.3	13.3	11.6	8.5	4-10
ESR	15	19	12	8	4	7	0-5
CRP (mg/L)	7	32	52	34	22	16	0-5
K (mmol/L)	6.3	6.4	5.2	-	3.7	3.3	3.4-4.9
Na (mmol/L)	122	126	127	129	138	139	133-146
Ca (mmol/L)	2.40	2.41	1.15	-	2.08	1.08	1.15-1.33
Urea (mmol/L)	6	6.3	15.3	11.7	11.5	10.6	2.5-7.8
Cr (umol/L)	56	61	89	62	64	66	45-84
eGFR (mL/minute)	89	88	85	89	>90	>90	>90
Total bilirubin (umol/L)	21	25	-	-	28	13	0-21
ALT (unit/L)	956	897	-	-	657	516	0-33
ALP (unit/L)	181	198	-	-	156	126	30-130
INR	1.6	1.5	1.5	1.4	-	1.6	0.9-1.1

During the current admission, two sets of blood cultures were negative. A computed tomography (CT) scan of the brain did not demonstrate any acute intracranial bleeding or thrombosis; however, a brain MRI confirmed the small micro-hemorrhages of the temporal region. Echocardiography on current admission demonstrated an echogenic structure size of 1.5 cm attached on the annulus and septal leaflet, thickened anteroposterior leaflets, and severe torrential tricuspid regurgitation with TV gradient of 4 mL/second. Echocardiogram also showed a severely dilated right ventricle (RV) (RV basal size: 5.3 cm, RV mid: 4.6 cm, RV length: 9.3 cm) and severely impaired biventricular function and left ventricular ejection fraction (LVEF) of 28% (Videos 1-4).

**Video 1:** Transthoracic echocardiogram shows biventricular dysfunction and echogenic structure on the tricuspid valve

**Video 2:** Four-chamber transthoracic echocardiogram color Doppler shows severe tricuspid regurgitation

**Video 3:** Transthoracic echocardiogram four-chamber view shows severe tricuspid regurgitation

**Video 4:** Transthoracic echocardiogram apical three-chamber view shows moderate mitral regurgitation

An ultrasound of the abdomen showed fatty liver and congested hepatic veins suggestive of congestion and splenomegaly. As a result, the patient was commenced on IV antibiotics, bisoprolol, digoxin, IV furosemide, and apixaban. She remained to be in atrial fibrillation with a rapid ventricular response with poor response to rate-controlled therapy and was given intravenous amiodarone followed by short-term oral amiodarone for rate control. Transoesophageal echocardiography demonstrated TV partial annuloplasty ring, septal leaflet appeared flail, and lack of coaptation with other leaflets. It also showed severe torrential TR with a TV gradient of 4 mL/s, moderate to severe secondary mitral regurgitation (MR) depending on the atrial fibrillation rate, and severely impaired biventricular function and underfilled LV (Videos 5-7).

**Video 5:** Transesophageal echocardiogram mid-esophageal view shows severe tricuspid regurgitation

**Video 6:** Transesophageal echocardiogram color flow shows moderate to severe mitral regurgitation

**Video 7:** Transesophageal echocardiogram shows moderate to severe mitral regurgitation

The patient was declined for surgery following discussion in the multidisciplinary team meeting (MDT) due to high risk and poor prognosis, and a conservative approach was advised. Antibiotics were stopped as transoesophageal echocardiogram (TOE) did not show any evidence of IE. She was also reviewed by the drugs misuse management team and was commenced on methadone. She remained apyrexial and was switched over to oral furosemide from intravenous treatment. She remained on bisoprolol and digoxin for rate control, and apixaban was continued. She was discharged home after three weeks of hospital stay and has had several admissions since then for repeated heart failure.

## Discussion

Infective endocarditis is a multisystem disease. It is a severe form of valve disease still associated with high mortality (15%-30% in-hospital mortality). Tricuspid valve endocarditis is most common in patients with intravenous drug use. Large vegetation and persistent bacteremia with septic embolic phenomena are the most common indication for surgery [6]. It exhibits a range of clinical signs and symptoms, including fever, muscle wasting, anorexia, nausea, vomiting, disorientation, breathing difficulty, chest discomfort, and embolic characteristics, including splinter hemorrhage, Roth spots on fundoscopy, Osler nodes, and Janeway lesions.

The diagnosis of IE can be extremely challenging in a subgroup of patients, such as patients with prosthetic valves, intracardiac devices, and blood culture-negative IE. Duke’s criteria are helpful for categorizing IE, but their utility is constrained in certain subgroups. The foundation of IE diagnosis is blood cultures and echocardiography. A transthoracic echocardiogram (TTE) must be done initially, but in the majority of cases of suspected or confirmed IE, both TTE and TEE should be done [7,8]. Choosing the best therapeutic approach requires careful consideration of the prognostic assessment upon admission. Simple clinical, microbiological, and echocardiographic characteristics can be used to conduct it [8]. The most frequent side effects are septic pulmonary embolism, abscess development, and valvular insufficiency [5]. According to a recent review, right-sided IE patients experienced an average of 1.6 sequelae, with valvular insufficiency, embolic events, and abscess formation being the most frequent [9].

The cornerstone of treatment for right-sided IE affecting the TV is intravenous antibiotics. Surgical procedures include removing vegetation, completely removing vegetation, and repairing diseased tissue and valves. When possible, prosthetic materials (such as valve replacement) should not be used [3,10]. Additionally, there are “prosthetic” and “non-prosthetic” surgical procedures (such as tricuspid valve replacement and ring annuloplasty).

Jiang et al. (2011) reported the incidence of TR in 11 patients from 35 patients who had tricuspid valve surgery for IE. One patient from this group died due to uncontrollable sepsis and multi-organ failure. Two patients from this group required mechanical ventilation, and three patients required renal replacement therapy for acute renal failure. Twenty-three patients had no valvular incompetence, whereas seven patients had mild TR, three patients had moderate TR, and one patient had severe tricuspid regurgitation [10]. Only one patient had severe TR requiring tricuspid valve replacement seven years later after the first operation. At 10 years follow-up, 19 patients had trivial or no TR, five patients had mild, two patients had moderate, and one patient had severe regurgitation. However, none of these patients required surgery for reinfection in the tricuspid valve. Aggressive debridement of the valve is required to prevent the spread of the infection to the circulation, and if the infection is confined to the valve, the vegetation should be removed where possible. Similarly, defects that developed due to IE should be repaired with autologous or homologous pericardial patches, and whenever valve repair is attempted, the use of artificial material should be avoided [10,11]. Most patients with right-sided IE benefit from intravenous antibiotics; however, 20% of patients require surgery [12].

## Conclusions

Tricuspid regurgitation is the most common presentation in patients with intravenous drug use. The management of tricuspid valve endocarditis and severe tricuspid regurgitation is challenging. The mortality risk remains quite high in this group, and tricuspid regurgitation following prosthetic tricuspid valve replacement can be challenging to manage. Infective endocarditis can lead to biventricular heart failure along with embolic ramifications. Early detection and prompt intervention can intercept further damage to the valve and related complications.
